# Identification of immune infiltration-related biomarkers in carotid atherosclerotic plaques

**DOI:** 10.1038/s41598-023-40530-w

**Published:** 2023-08-29

**Authors:** Kai Zheng, Wentao Yang, Shengxing Wang, Mingsheng Sun, Zhenyi Jin, Wangde Zhang, Hualiang Ren, Chunmin Li

**Affiliations:** Department of Vascular Surgery, Beijing Chaoyang Hospital, Capital Medical University, Beijing, China

**Keywords:** Biological techniques, Biotechnology, Cell biology, Computational biology and bioinformatics, Genetics, Immunology

## Abstract

Atherosclerosis is a chronic lipid-driven inflammatory response of the innate and adaptive immune systems, and it is responsible for several cardiovascular ischemic events. The present study aimed to determine immune infiltration-related biomarkers in carotid atherosclerotic plaques (CAPs). Gene expression profiles of CAPs were extracted from the Gene Expression Omnibus database. Differentially expressed genes (DEGs) between the CAPs and control groups were screened by the “limma” package in R software. Immune cell infiltration between the CAPs and control groups was evaluated by the single sample gene set enrichment analysis. Key infiltrating immune cells in the CAPs group were screened by the Wilcoxon test and least absolute shrinkage and selection operator regression. The weighted gene co-expression network analysis was used to identify immune cell-related genes. Hub genes were identified by the protein–protein interaction (PPI) network. Receiver operating characteristic curve analysis was performed to assess the gene’s ability to differentiate between the CAPs and control groups. Finally, we constructed a miRNA-gene-transcription factor network of hub genes by using the ENCODE database. Eleven different types of immune infiltration-related cells were identified between the CAPs and control groups. A total of 1,586 differentially expressed immunity-related genes were obtained through intersection between DEGs and immune-related genes. Twenty hub genes were screened through the PPI network. Eventually, 7 genes (*BTK*, *LYN*, *PTPN11*, *CD163*, *CD4*, *ITGAL*, and *ITGB7*) were identified as the hub genes of CAPs, and these genes may serve as the estimable drug targets for patients with CAPs.

## Introduction

Chronic accumulation of lipid-driven plaques in the subendothelial intima of arteries eventually leads to significant stenosis of the lumen, insufficient supply of blood flow, and critical hypoxia of the tissue^1^. Cardiovascular diseases, peripheral artery diseases, and cerebrovascular diseases are commonly caused by atherosclerosis. Carotid atherosclerotic plaques (CAPs) are also the most common underlying pathology of ischemic cerebral stroke. Previous studies have greatly enhanced our understanding of the etiology of atherosclerosis. However, there is still lack of clinical practice to translate fundamental science to patient bedside^2^. Therefore, there is a need to filtrate hub genes to differentiate patients with CAPs from controls.

The key mechanism of atherosclerosis development is an immune response to lipid-driven inflammation in the subendothelial intimal layer of arteries. Inflammatory macrophages and foam cell formation during plaque progression play particularly important roles in atherogenesis, and they have been extensively reviewed previously^1,3^. Innate and adaptive immune cells contribute to the development of arterial chronic inflammation^4^. Abnormal distribution and incommensurate types of immune cells are also associated with atherogenesis^5^. Therefore, the exploration of various alterations of immune cells in atherogenesis could offer novel insights into the etiology, diagnosis, and treatment of CAPs.

High-throughput sequencing (HTS) technologies, for example, microarray, RNA sequencing, and single cell sequencing, generate abundant gene expression data which are appropriate for studying the distribution of immune cells in lesion tissues^6,7^. The identification of types, composition, and functional states of cells in atherogenesis is crucial to understand the cellular contribution^8–10^. Gene expression profiling; for example, microarray and RNA sequencing, could be used to identify differentially expressed genes (DEGs) and signaling pathways that participate in atherogenesis^11,12^. Through combination with sequencing techniques, bioinformatics analysis could elucidate the relationships between DEGs and atherogenesis and illustrate the interaction network of hub genes, heterogeneous nuclear RNA (hnRNA), transcriptional factors (TFs), microRNAs (miRNAs), and proteins^13,14^.

In the present study, the microarray data were downloaded from Gene Expression Omnibus (GEO) datasets, and the single sample gene set enrichment analysis (ssGSEA) algorithm was used to evaluate immune cell abundance and types between the CAPs and control groups. We also identified the immune infiltration-related hub genes in patients with CAPs.

## Methods

### Data acquisition

GEO is an international public repository for microarray and sequence functional genomic datasets deposited by researchers. The gene expression datasets GSE43292 and GSE100927 were used for the differential gene analysis between the CAPs and control groups. A total of 32 CAPs obtained from endarterectomy tissue and 32 macroscopically intact carotid tissues adjacent to the CAPs were included in GSE43292. A total of 29 CAPs and 12 normal carotid artery tissues were included in GSE100927. The GSE43292 dataset was used as the discovery set, while the GSE100927 dataset was used for validation.

### Data preprocessing and screening of DEGs

The “limma” package in R software^15^ was used to conduct the differential expression of mRNAs. The adjusted *P* value, which was adjusted by using the Benjamini and Hochberg (BH) method, was used to correct false-positive results in GSE43292. All DEGs (adjusted *p* value < 0.001) between the CAPs and control groups were selected for further analysis.

### Functional and pathway enrichment analysis

To assess the potential biological functions of DEGs, Gene Ontology (GO) and Kyoto Encyclopedia of Genes and Genomes (KEGG) pathway enrichment analysis were performed by using the “clusterProfiler” package^16,17^. Functional categories with an adjusted *P* value of < 0.05 (adjusted by the Benjamini and Hochberg (BH) method) were considered as significant pathways.

### Analysis of immune cell characteristics

The infiltration level of immune cells (activated B cells, activated CD4 + T cells, activated CD8 + T cells, activated DCs, CD56bright natural killer cells, CD56dim natural killer cells, central memory CD4 + T cells, central memory CD8 + T cells, effector memory CD4 + T cells, effector memory CD8 + T cells, eosinophils, gamma-delta T cells, immature B cells, immature DCs, macrophages, mast cells, myeloid-derived suppressor cells (MDSCs), memory B cells, monocytes, natural killer cells, natural killer T cells, neutrophils, plasmacytoid DCs, regulatory T cells, T follicular helper cells, Type 1 T helper cells, Type 17 T helper cells, and Type 2 T helper cells) was determined by the ssGSEA algorithm in the “GSVA” package of R software^18^. The background gene set was collected from the previous literature^18^.The ssGSEA algorithm defined a score representing the degree of absolute enrichment of different immune cell geneset in each sample. The differences in the immune cell infiltration between the CAPs and normal groups were analyzed by the Wilcoxon test (*P* < 0.05). The signature immune cells that could be used to differentiate between disease and control samples were screened using the least absolute shrinkage and selection operator (LASSO) regression analysis. The characteristic of LASSO regression is that when the generalized linear model is established, the requirement for data is extremely low, so it is widely applied. By carrying out regression punishment on all variables, the coefficient of relatively unimportant variables becomes 0, which is excluded from the modeling. Then, independent variables with great influence on dependent variables are selected and the corresponding regression coefficient is calculated. Finally, important features are screened out. The intersecting immune cells of the two methods were extracted as the key immune cells of CAPs for the subsequent study.

### Weighted gene co-expression network construction and analysis

The R package “WGCNA” was used to find key infiltrating immune cell-related modules and genes^19^. First, hierarchical clustering was performed on the CAPs to detect and eliminate outliers. Next, we used the pickSoftThreshold function to find a soft threshold power β in accordance with standard scale-free networks. The adjacencies between all filtered genes were calculated using the power adjacent function to Pearson’s correlation matrix to transform data into a topological overlap matrix (TOM), and the corresponding dissimilarity (1-TOM) was calculated. The dissimilarity of module eigengenes was then calculated to further analyze the module.

### Construction of the PPI network and identification of hub genes

The DEIRGs were obtained by intersecting module genes and DEGs. The PPI network of the DEIRGs was constructed using the STRING database^20^ with the minimum required interaction score of 0.7 (highest confidence). Subsequently, the core module of the PPI network was screened by the Cytoscape software plugin Molecular Complex Detection (MCODE)^21^ with default parameters as follows: degree cutoff = 2, node score cutoff = 0.2, and k-score = 2. The genes in the core module were considered as the hub genes.

### Receiver operating characteristic curve analysis of hub genes

Receiver operating characteristic (ROC) curve analysis is a well-established method to assess how well a marker is capable of discriminating between individuals who experience disease onset and individuals who do not. The ROC curve was plotted, and the area under the curve (AUC) was calculated with the “pROC” package of R software^22^ to evaluate the capability of the selected hub genes to differentiate between the CAPs and control groups.

### Expression correlation, functional similarity, and immune cell correlation analysis of the diagnostic genes

Correlation coefficients between the genes were calculated by Spearman’s correlation coefficient analysis using the “corrplot” package of R software^23^^.^ Moreover, the functional similarity among the genes was determined using the geometric mean of semantic similarities in cellular components (CC) and molecular functions (MF) through the “GOSemSim” package^24^. To further investigate the relationship between hub genes and immune cells, we also analyzed the correlation between hub genes and differentially infiltrating immune cells.

### Disease ontology enrichment analysis and identification of potential drugs

Disease ontology (DO) annotates human genes in the context of the disease. The “DOSE” package of R software^25^ was used to analyze the DO enrichment of the diagnostic genes. DO terms with significant enrichment were screened on the basis of the threshold adjusted *P* value of < 0.05. The Drug Gene Interaction Database (DGIdb) is a resource for drug-gene interaction^26^. We searched the DGIdb database to predict potential drugs or molecular compounds that interacted with the diagnostic genes and visualized the drug-gene interaction network by the Cytoscape software.

### Construction of the regulatory network

miRNAs or TFs control gene expression through interaction with their target genes during the post-transcriptional or transcriptional stage. The concrete implementation process was achieved through the miRnet database (https://www.mirnet.ca/miRNet/home.xhtml), which could select the background database when making TF predictions. So the ENCODE database was used to predict miRNAs and TFs that interacted with the hub genes^21^. We visualized the miRNA-gene-TF network by using Cytoscape software.

## Results

### Identification and enrichment analysis of DEGs

**Figure 1:**
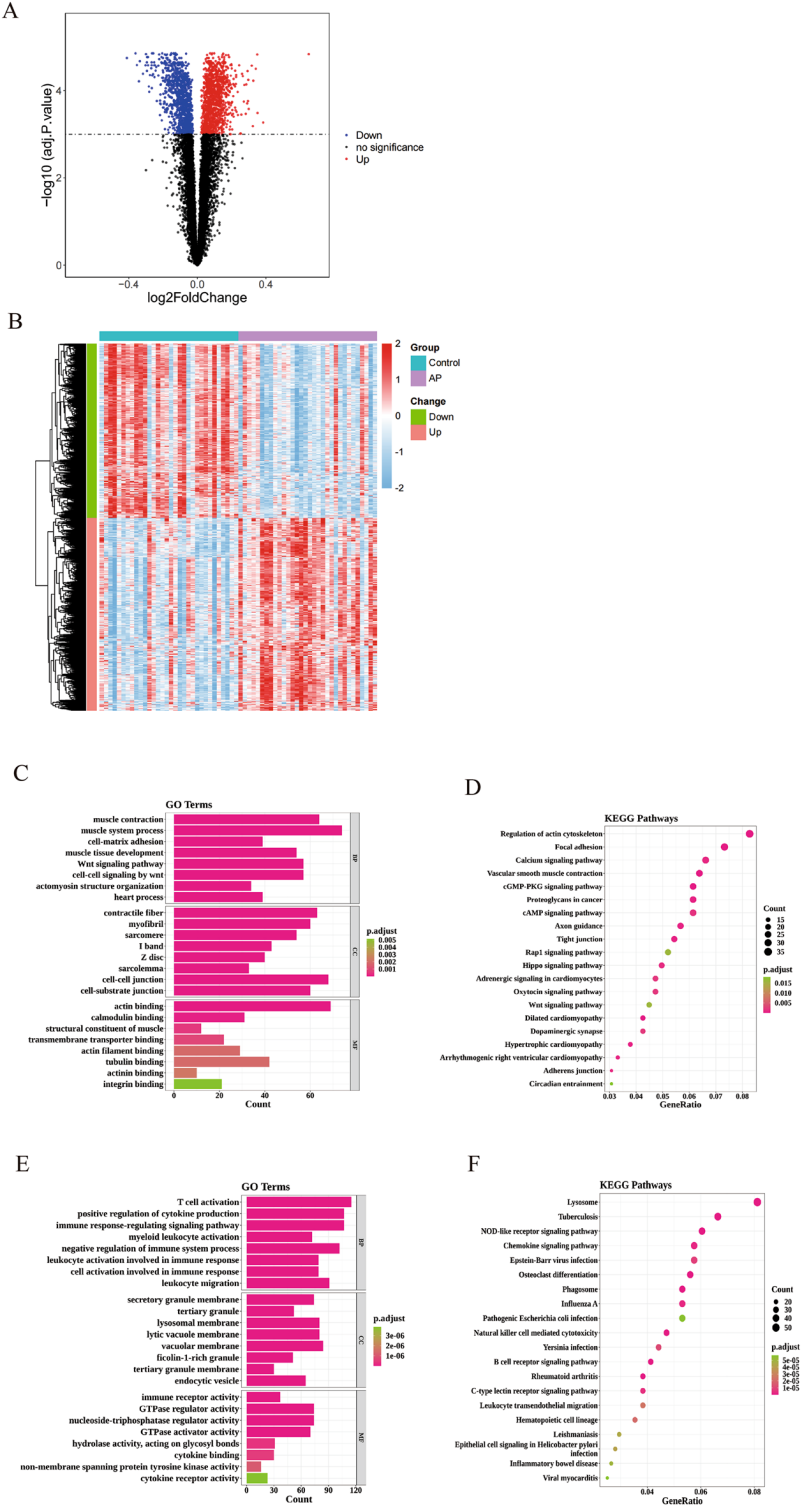
Identification and Enrichment Analysis of DEGs. (**A**) Volcano map of DEGs. The blue dots represent genes that were significantly downregulated in the CAPs, the red dots represent genes that were significantly upregulated in CAPs. (**B**) Heat map of DEGs. The ordinate represents the samples, and the abscissa represents the DEGs. The heat maps were produced by pheatmap (1.0.12 Kolde R (2019). _pheatmap: Pretty Heatmaps_. R package version 1.0.12, < https://CRAN.R-project.org/package=pheatmap > .) (**C**) GO biological function enrichment analysis of down-regulated genes. (**D**) KEGG pathway enrichment analysis of down-regulated genes. (E) GO biological function enrichment analysis of up-regulated genes. (**F**) KEGG pathway enrichment analysis of down-regulated genes.

A total of 2,378 DEGs (1,248 upregulated and 1,130 downregulated) were identified in CAPs when compared with normal groups (Fig. 1A). The expression of DEGs in patients with CAPs and normal groups is shown in a heatmap (Fig. 1B). In order to explore the potential molecular mechanisms of these DEGs, we performed GO and KEGG enrichment analyses.

Regarding the downregulated genes, GO analysis revealed that the primary functional categories in the biological processes (BP) were “muscle system process,” “muscle contraction,” and “Wnt tissue development.” For cellular component (CC), the major enriched GO terms were “cell–cell junction,” “contractile fiber,” and “myofibril.” The most enriched molecular function (MF) terms were “actin binding,” “calmodulin,” and “structural constituent of muscle” (Fig. 1C). The KEGG pathway enrichment analysis indicated that the DEGs were mainly involved in “Regulation of actin cytoskeleton,” “Focal adhesion,” “Calcium signaling pathway,” and “Vascular smooth muscle contraction” (Fig. 1D).

Regarding the upregulated genes, GO analysis revealed that the primary functional categories in BP were “T cell activation,” “positive regulation of cytokine production,” and “immune response-regulating signaling pathway.” For cellular component (CC), the major enriched GO terms were “vacuolar membrane,” “lytic vacuole membrane,” and “lysosomal membrane.” The most enriched MF terms were “GTPase regulator activity,” “nucleoside-triphosphatase regulator activity,” and “GTPase activator activity” (Fig. 1E). The KEGG pathway enrichment analysis indicated that the DEGs were mainly involved in “Lysosome,” “Tuberculosis,” and “Chemokine signaling pathway” (Fig. 1F).

### Infiltrating immune cell analysis

**Figure 2:**
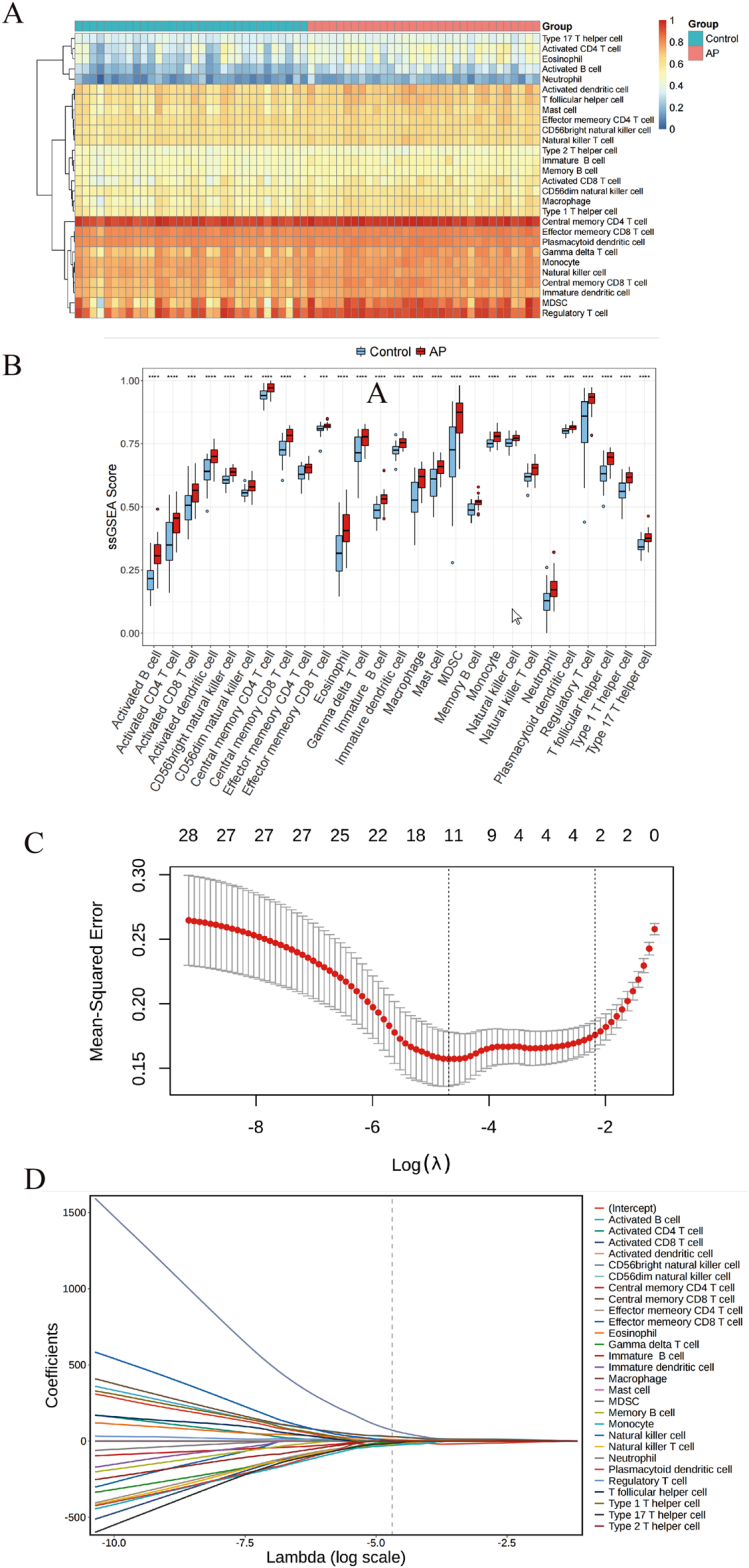
Infiltrating Immune Cell Analysis. (**A**) Heat map of immune cell infiltration. The abscissa represents the sample and the ordinate represents the immune cell type (N = 28). The color represents the amount (or activity) of immune cells in each sample. The heat maps were produced by pheatmap (1.0.12 Kolde R (2019). _pheatmap: Pretty Heatmaps_. R package version 1.0.12, < https://CRAN.R-project.org/package=pheatmap > .) (**B**) Box plot of immune cell infiltration between CAPs and control groups. The abscissa represents immune cell type and the ordinate represents ssGSEA Score. The blue box represents the control groups, and the red box represents the CAPs. *p < 0.05, **p < 0.01, ***p < 0.001, ****p < 0.0001. The graph shows only the 27 types of immune cells are significantly different. (**C**)(**D**) Differential immune cells were identified by LASSO algorithm.

Since the immune microenvironment is closely related to the onset and progression of disease, we performed immune infiltration analysis. Firstly, GSVA package was used to calculate ssGSEA scores of 28 different types of immune cells in each sample. Next, we compared the infiltration of immune cells between CAPs and normal groups. Figure 2A shows the infiltration level of the immune cells in CAPs and normal groups. To compare scores for the absolute enrichment of different immune cell gene sets in each sample, the results of the Wilcoxon test are presented in Fig. 2B, which showed 27 types of immune cells with a *P* value of < 0.05. Subsequently, LASSO logistic regression analysis was conducted by R package glmnet to compare characteristic immune cells between AP and normal samples. The results of LASSO were presented in Fig. 2C, D, which showed 11 types of immune cells that could be used as signature cells of CAPs. The differential immune cells from the Wilcoxon test and signature immune cells from LASSO regression were intersected, and 11 immune cells were obtained and used as the key immune cells of CAPs; these included activated B cells, activated CD8 + T cells, CD56bright natural killer cells, central memory CD8 + T cells, effector memory CD4 + T cells, effector memory CD8 + T cells, eosinophils, gamma delta T cells, memory B cells, monocytes, and natural killer T cells. These were significantly higher in CAPs than in normal groups.

### Discovery of the immune-related modules and genes

**Figure 3:**
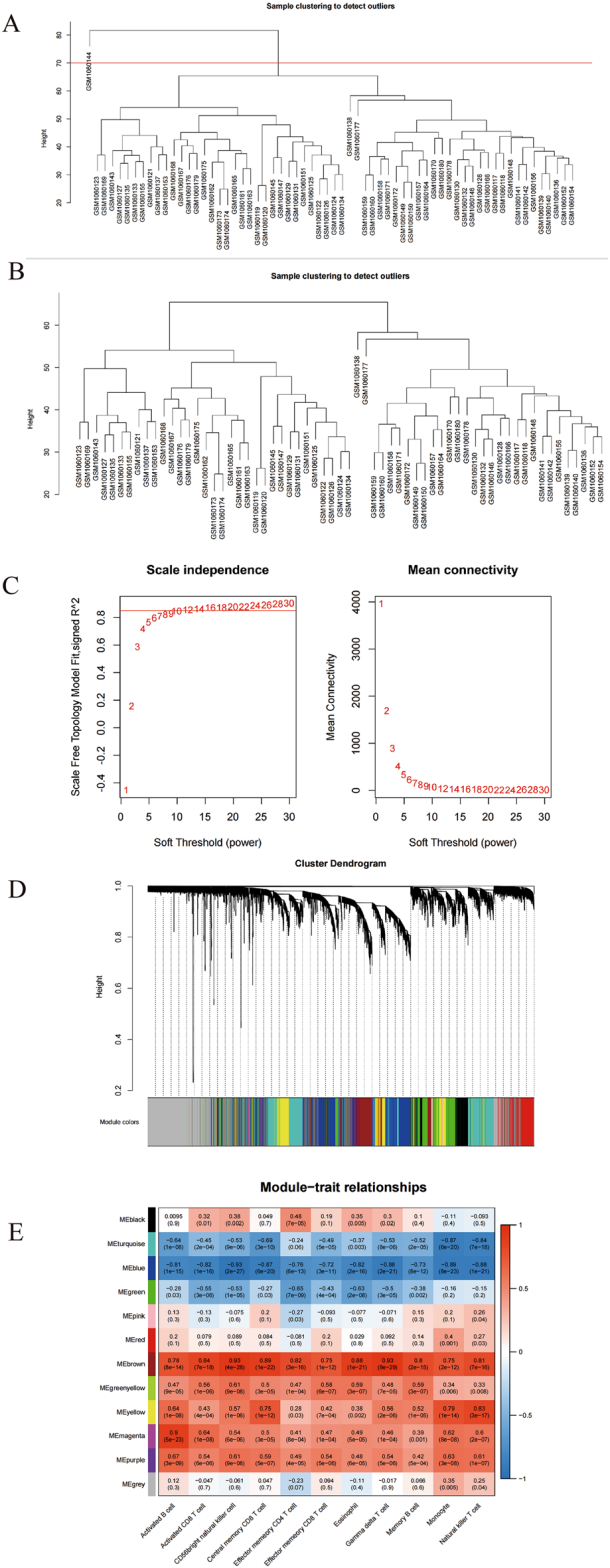
Discovery of the Immune-Related Modules and Genes. (**A**) Sample clustering tree. The total number of samples was N = 64, and the outlier sample GSM1060144 needed to be removed. (**B**) Sample clustering tree after removing outlier samples. The total sample number is N = 63. (**C**) Filtering the soft threshold. Set the soft threshold to 12. (**D**) Hierarchical clustering tree. The upper part of the figure represents the clustering of genes, the lower part represents gene modules, forming a total of 12 modules, and the gray module represents genes not classified into modules. (**E**) Module—Trait correlation heat map. The values in the squares represent the Pearson correlation coefficients and corresponding *p* values calculated pairwise between traits and modules. The heat maps were produced by WGCNA 1.71 Langfelder P and Horvath S, WGCNA: an R package for weighted correlation network analysis. BMC Bioinformatics 2008, 9:559 https://doi.org/10.1186/1471-2105-9-559, http://horvath.genetics.ucla.edu/html/CoexpressionNetwork/Rpackages/WGCNA/.

To screen genes associated with the key infiltrating immune cells, the co-expression network was constructed using weighted gene co-expression network analysis (WGCNA). First, the Euclidean distance of gene expression was used to perform hierarchical clustering of samples, and the outlier data were excluded (Fig. 3A, B). Next, by using the soft-thresholding power of 12, a total of 12 modules were identified (Fig. 3C, D). To further evaluate the relationship between modules and infiltrating immune cells, Module-Trait correlation heat maps was made (Fig. 3E). We observed that the correlations between both blue and brown modules, and 11 differential immune cells were greater than that for other modules (Fig. 3E). Consequently, 3,719 module genes in the blue and brown modules were considered immune-related genes for further studies.

### Identification of hub genes by the PPI network

**Figure 4:**
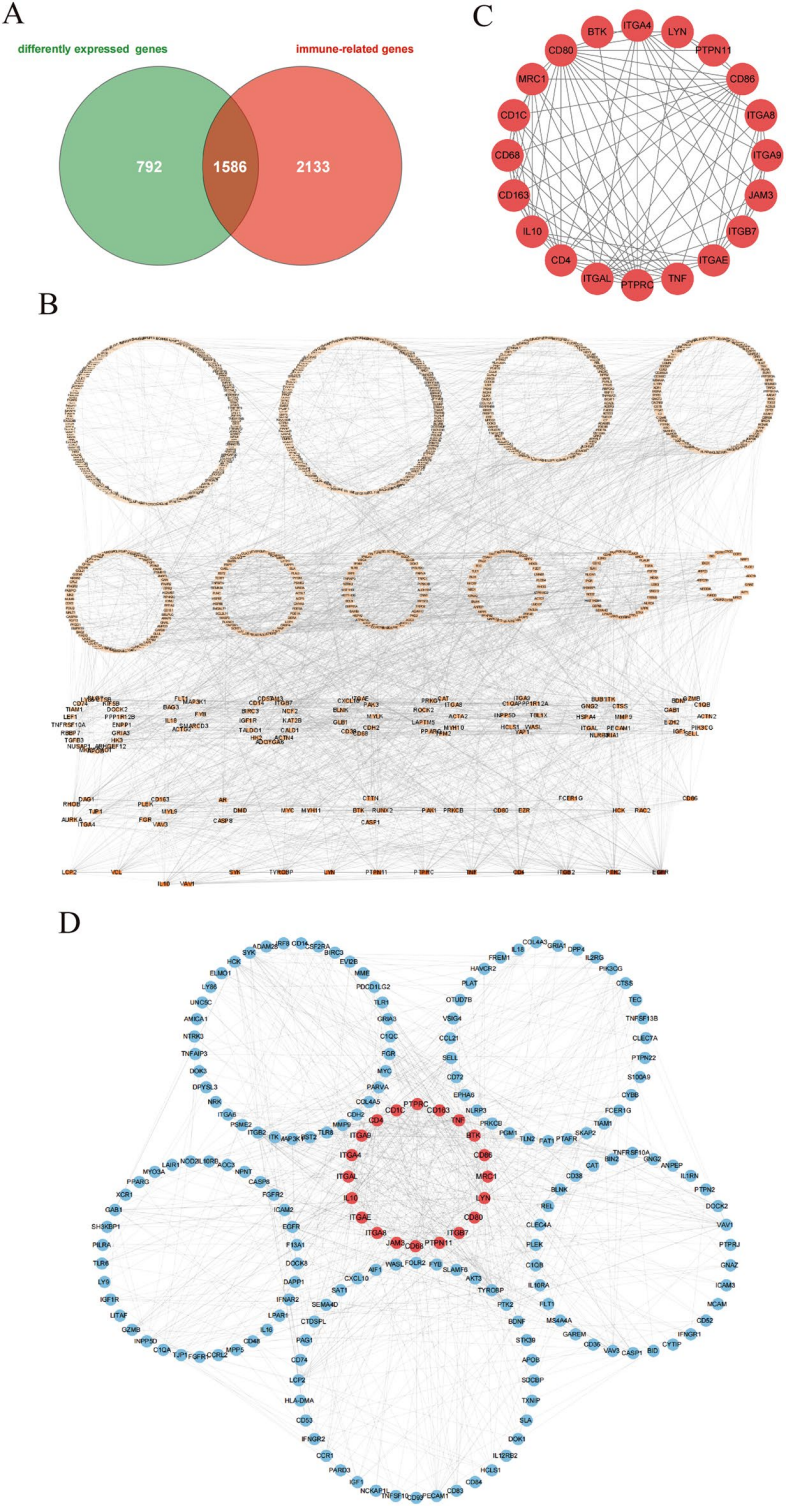
Identification of Hub Genes by PPI Network. (**A**) Venn diagram of intersection of DEGs and immune-related genes. The green area represents DEGs, the red area represents immune-related genes, and the overlap represents DEIRGs (N = 1586). (**B**) Protein interaction network. Nodes represent differentially expressed circadian related proteins. The line between nodes indicates that there is an interaction between them. (**C**) MCODE1 score = 9.368. Select all genes in this module (Top1) as key genes (N = 20). (**D**) The PPI of 20 hub genes.

The obtained 3,719 immune-related genes and 2,378 DEGs were intersected, and a total of 1,586 DEIRGs were obtained (Fig. 4A). The PPI was analyzed with 1,586 DEIRGs using the STRING database (Fig. 4B). The entire PPI network was analyzed using MCODE, following which the genes in the core module were chosen as hub genes (Fig. 4C). Finally, 20 hub genes, namely *ITGB7*, *PTPN11*, *CD68*, *JAM3*, *ITGA8*, *ITGAE*, *IL10*, *ITGAL*, *ITGA4*, *ITGA9*, *CD4*, *CD1C*, *PTPRC*, *CD163*, *TNF*, *BTK*, *CD86, MRC1*, *LYN*, and *CD80*, were identified. The PPI of the 20 hub genes is shown in Fig. 4D.

### Evaluation and validation of the diagnostic performance of the hub genes

**Figure 5:**
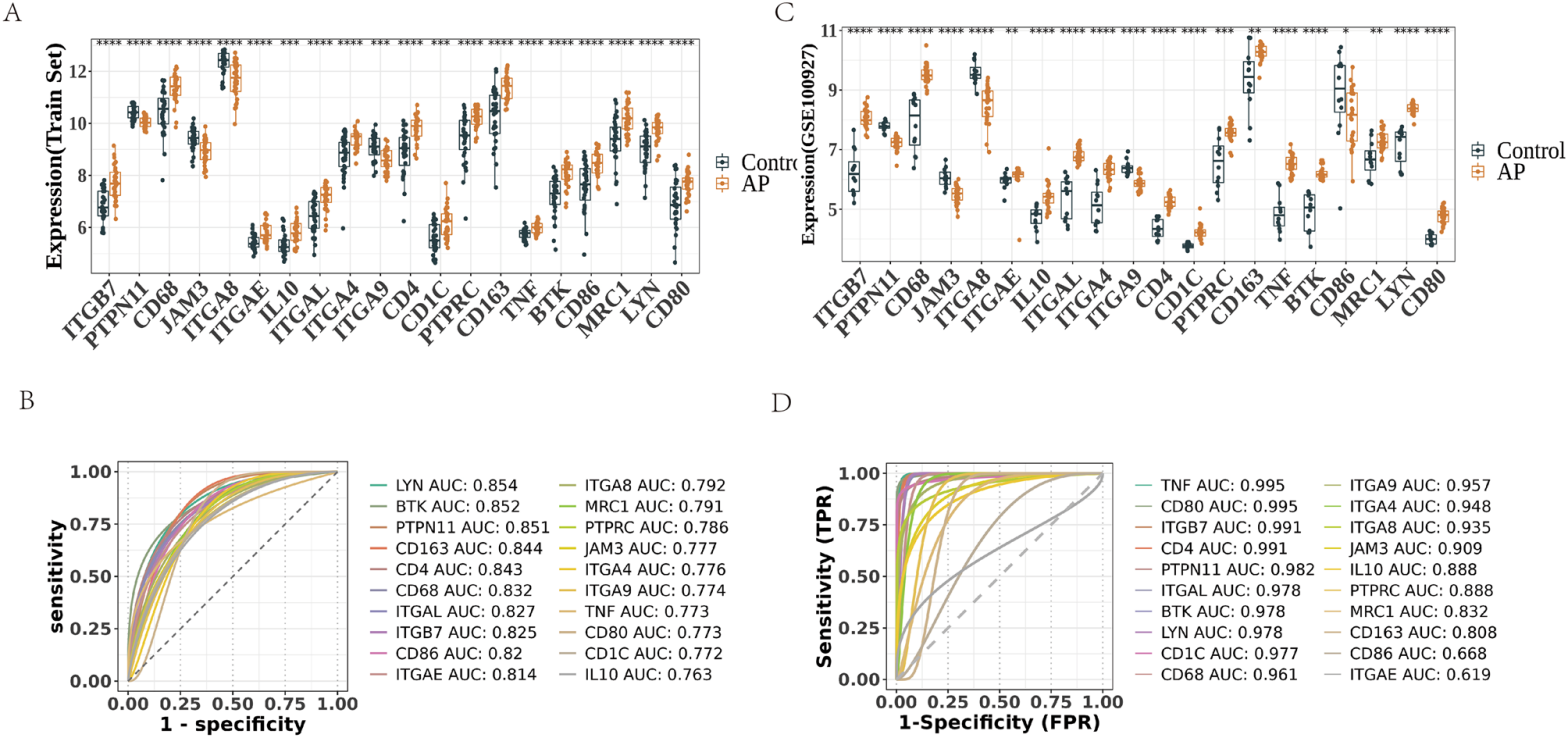
Verifcation of Hub Genes. (**A**) Boxplot of differential expression hub genes between CAPs and control groups in discovery dataset. (**B**) ROC curve validation based on the expression of key genes in discovery dataset. The AUC value represents the area under the curve. (**C**) Boxplot of differential expression hub genes between CAPs and control groups in validation dataset. (**D**) ROC curve validation based on the expression of key genes in validation dataset.

First, the expression levels of the hub genes were visualized. The expression level of *ITGB7*, *CD68*, *ITGAE*, *IL10*, *ITGAL*, *ITGA4*, *CD4*, *CD1C*, *PTPRC*, *CD163*, *TNF*, *BTK*, *CD86*, *MRC1*, *LYN*, and *CD80* was higher, and the expression level of *PTPN11*, *JAM3*, *ITGA8*, and *ITGA9* was lower in patients with CAPs (Fig. 5A). Next, we constructed receiver operating characteristic (ROC) curves for the hub genes and calculated the corresponding area under the curve (AUC) values (Fig. 5B). The results showed that the AUCs of the hub genes (*BTK*, *LYN*, *PTPN11*, *CD163*, *CD4*, *CD68*, *ITGAL*, *ITGB7*, and *ITGAE*) were greater than 0.8. The expression levels of the hub genes were further verified in the validation dataset GSE100927 (Fig. 5C). There were 19 hub genes (except *CD86*) with expression trends similar to those of the discovery set. As shown in Fig. 5D, the AUC values of the 18 hub genes (except *CD86* and *ITGAE*) were also greater than 0.7. Thus, the validated 18 hub genes, namely *ITGB7*, *PTPN11*, *JAM3*, *ITGA8*, *IL10*, *ITGAL*, *ITGA4*, *ITGA9*, *CD4*, *CD1C*, *PTPRC*, *CD163*, *TNF*, *BTK*, *CD68*, *MRC1*, *LYN*, and *CD80*, were selected, and these genes were found to have the ability to differentiate between the CAPs and control groups.

### Expression correlation, functional similarity, and immune cell correlation analysis of the diagnostic genes

**Figure 6:**
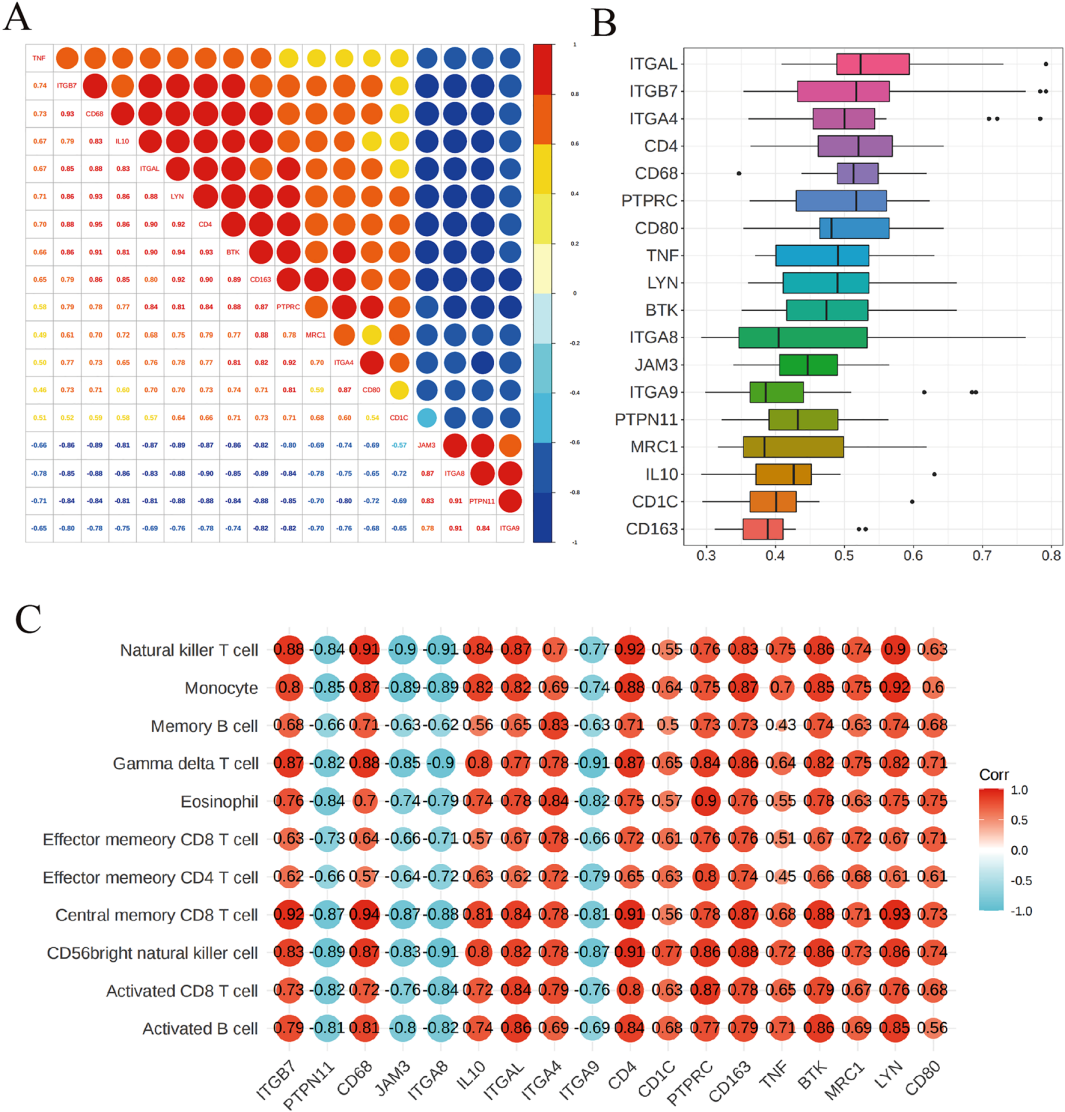
Expression Correlation, Functional Similarity and Immune Cell Correlation Analysis of the Hub Genes. (**A**) Hub gene expression correlation coefficient diagram. (**B**) Functional similarity of hub genes. The abscissa represents the gene functional similarity score, the ordinate represents the gene name, and different colored bins represent different genes. (**C**) Heat map of association between hub genes and differential immune infiltrating cells. The heat maps were produced by ggcorrplot 0.1.4 Kassambara A (2022). _ggcorrplot: Visualization of a Correlation Matrix using 'ggplot2'_. R package version 0.1.4, https://CRAN.R-project.org/package=ggcorrplot.

To demonstrate the correlations among the hub genes, we made Fig. 6A. The genes *CD80*, *ITGA4*, *CD1C*, *PTPRC*, *MRC1*, *CD68*, *CD163*, *BTK*, *CD4*, *LYN*, *ITGAL*, *ITGB7*, *IL10*, and *TNF* were found to be positively correlated with each other. Moreover, the genes *ITGA9*, *PTPN11*, *JAM3*, and *ITGA8* were also positively correlated with each other. However, the genes *CD80*, *ITGA4*, *CD1C*, *PTPRC*, *MRC1*, *CD68*, *CD163*, *BTK*, *CD4*, *LYN*, *ITGAL*, *ITGB7*, *IL10*, and *TNF* were negatively correlated with the genes *ITGA9*, *PTPN11*, *JAM3*, and *ITGA8*. Comparing functional similarity of genes or gene products is an important content of life science research, which has a wide range of applications in functional prediction of biological macromolecules, gene clustering, biological network analysis and screening of disease-related genes. Calculating functional similarity between genes has become the basic work of bioinformatics research. Thus, we ranked the hub genes according to the average functional similarity. Figure 6B demonstrated that *ITGAL*, *ITGB7*, and *ITGA4* were the top three hub genes and might play key roles in CAPs.

Moreover, We need to clarify the association between the hub genes and differentially infiltrating immune cells. The result of the correlation analysis between the hub genes and differentially infiltrating immune cells indicated that the genes *ITGB7*, *IL10*, *ITGAL*, *ITGA4*, *CD4*, *CD1C*, *PTPRC*, *CD163*, *TNF*, *BTK*, *CD68*, *MRC1*, *LYN*, and *CD80* were positively correlated with differentially infiltrating immune cells, while the genes *PTPN11*, *JAM3*, *ITGA8*, and *ITGA9* were negatively correlated with differentially infiltrating immune cells (Fig. 6C).

### Disease ontology enrichment analysis of the hub genes and identification of potential drugs

**Figure 7:**
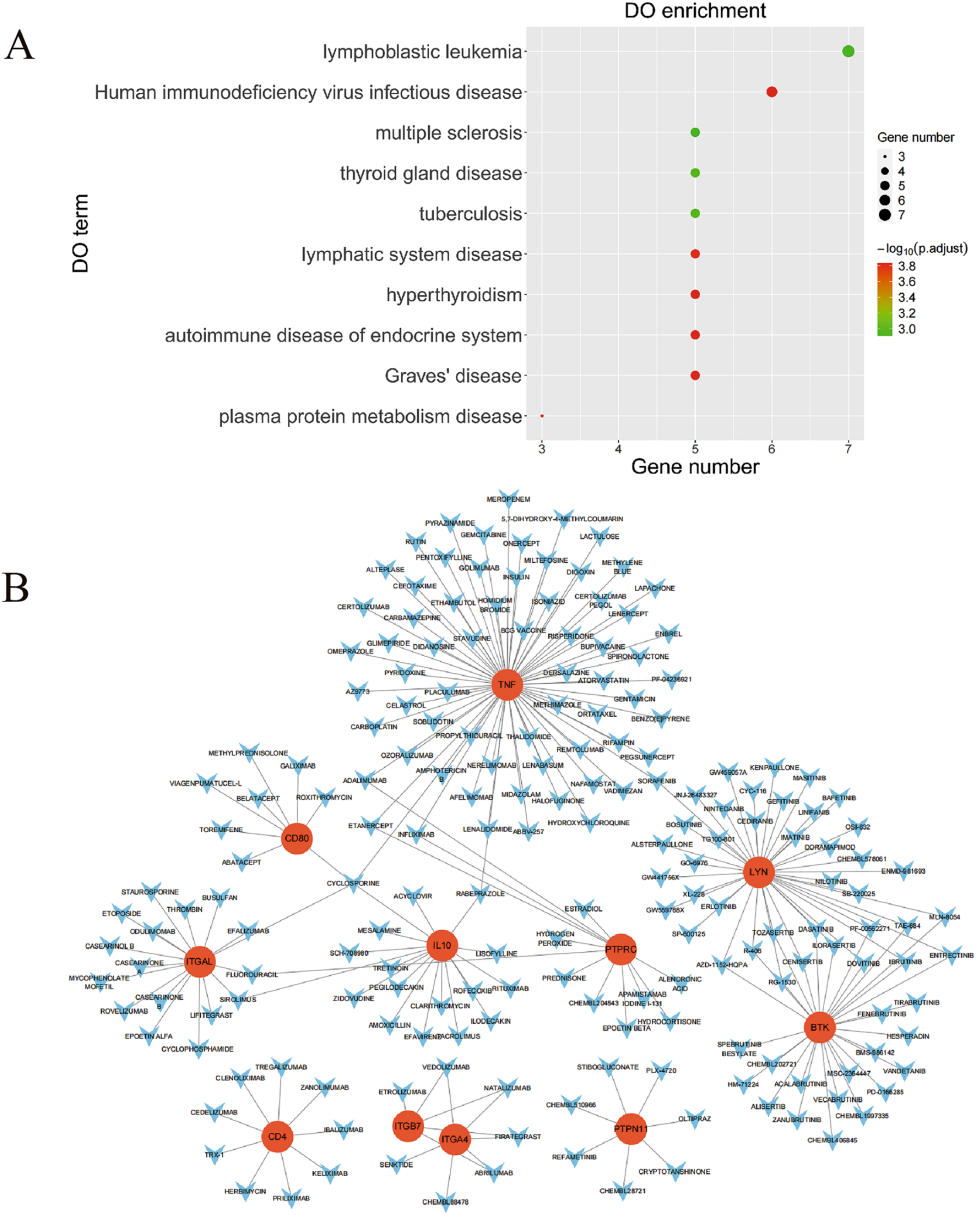
DO Enrichment Analysis of the Hub Genes and Identification of Potential Drugs. (**A**) DO enrichment bubble diagram. Only the top 10 disease types are shown. (**B**) Diagram of gene-drug interaction network, the line between the two indicates an interaction.

In order to explore the role of DEGs in other diseases, the Disease Ontology (DO) analysis showed that the hub genes were mainly enriched in “lymphoblastic leukemia,” “Human immunodeficiency virus infectious disease,” “multiple sclerosis,” and other terms (Fig. 7A). To search for potential therapeutic drugs, we performed an analysis on small molecule drug screening. As shown in the drug-gene interaction network (Fig. 7B), a total of 191 drugs or molecular compounds corresponding to 11 hub genes were retrieved from the DGIdb database. Cyclosporine could regulate 4 hub genes, namely *CD80*, *IL10*, *ITGAL*, and *TNF*. Additionally, 68 drugs or molecular compounds that included alteplase and atorvastatin regulated the gene *TNF*.

### Construction of the miRNA-gene-transcription factor regulatory network

**Figure 8:**
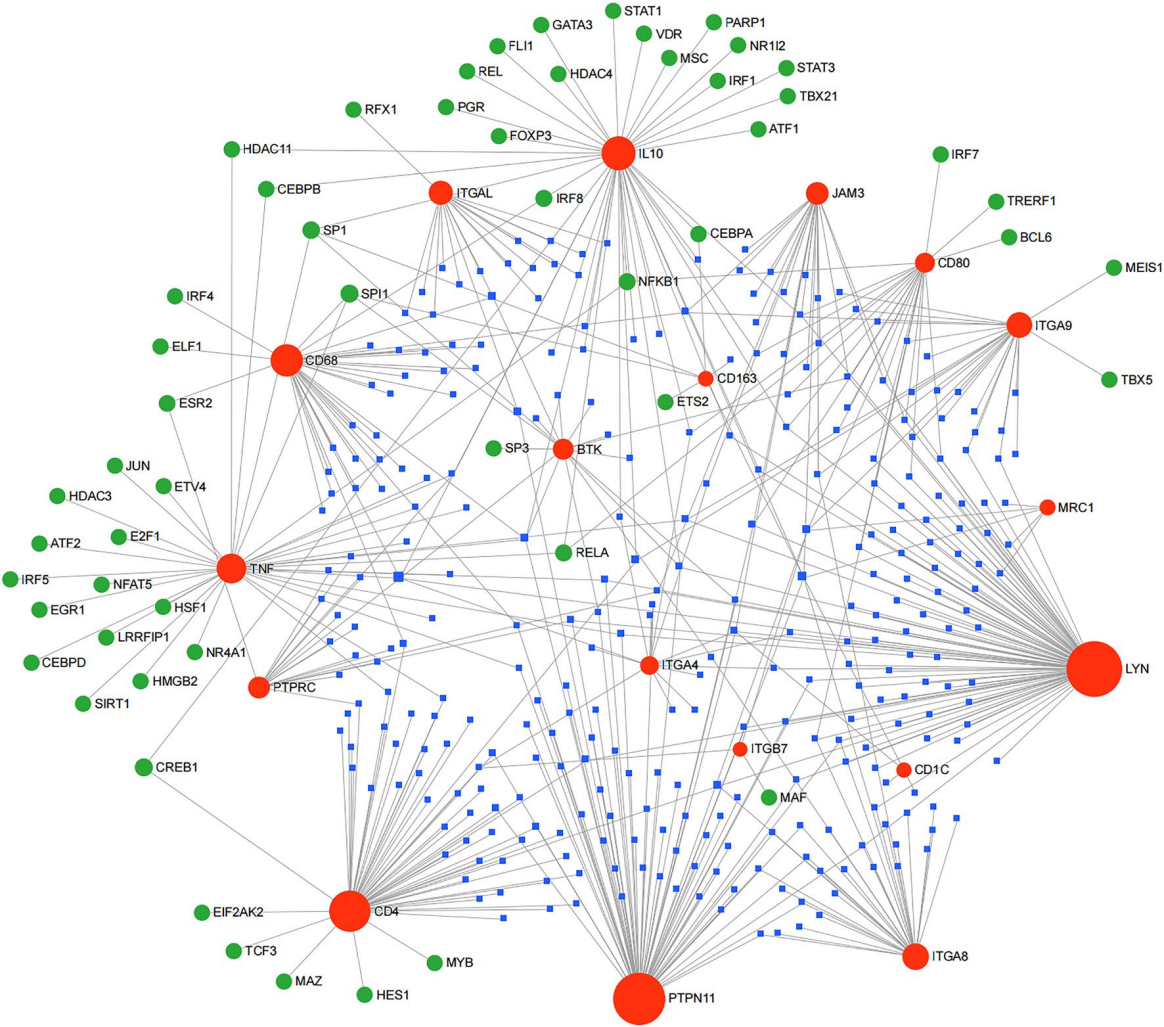
Construction of the miRNA-gene-TF Regulatory Network. Hub gene—TFs—mirNA regulatory network. The red area represents the hub gene, the green area represents the TFs, the purple area represents the miRNA, and lines indicates the existence of regulatory relationship.

To explore the pathways of hub genes, we constructed a miRNA-gene-transcription factor (TF) regulatory network by obtaining the regulatory interactions between miRNAs/TFs and the hub genes in the ENCODE database (Fig. 8). A total of 384 nodes (18 hub genes, 311 miRNAs, and 55 TFs) and 549 edges were included in the miRNA-gene-TF network. The top five miRNAs that regulated the largest number of hub genes were hsa-mir-155-5p, hsa-mir-7-5p, hsa-mir-1291, hsa-mir-328-3p, and hsa-mir-551b-3p. The five regulating genes *SP1*, *NFKB1*, *SPI1*, *RELA*, and *CREB1* ranked the highest among the TFs. *LYN*, *PTPN11*, and *TNF* were the top three hub genes targeted by miRNAs, and *IL10* and *TNF70* were the top two hub genes targeted by TFs.

## Discussion

CAPs is one of the leading causes of stroke^27^. The emboli formed by plaque rupture are mainly responsible for cerebral vascular occlusion. Therefore, screening of hub genes in patients at risk for stroke and determining the possible immunotherapeutic targets for CAPs are critical for preventing cerebrovascular adverse events^28^. To identify the hub genes of CAPs, we analyzed the co-regulated DEGs in the CAPs-related dataset GSE43292. By using integrated bioinformatics methods, 11 significantly different types of immune cells were identified between the CAPs and control groups, and 7 hub genes (*BTK, LYN, PTPN11, CD163, CD4, ITGAL, and ITGB7*) may serve as potential biomarkers to differentiate between CAPs and control samples. Although several immune-related signatures have been proposed in the literature, we propose for the first time novel immune infiltration-related biomarkers in carotid artery atherosclerosis.

Previous studies have screened DEGs though the GEO dataset^29–31^. Different methods and criteria have led to varying results for DEGs. Chen et al. screened 335 upregulated genes and 81 downregulated genes. Based on single gene GSEA, gene sets correlated with inflammation and immune response were highly upregulated in the high expression level group of *NCF2*, *IQPAG2*, and *CD86*^29^^.^ Liu et al. obtained 758 DEGs, and found that the genes, *ITGAM* and *ACTN2* showed high levels of expression in the PPI network^31^. Liu et al. identified 680 upregulated genes and 875 downregulated genes in plaque samples. The gene *IGFBP6* is downregulated in unstable CAPs; thus, it may be an important molecule in the formation of unstable plaques^30^. Ji et al. investigated changes in gut microbiota and plasma metabolites in patients with CAPs and control groups by using ribosomal DNA sequencing and metabolomics. Among 132 DEGs, the gene *FABP4* showed the highest |log2(fold change)|^32^. These studies identified only DEGs between stable and unstable plaques; however, our research identified DEGs between the CAPs and control groups, which is convincing and valuable.

Wang et al. identified key genes involved in the progression of CAPs by using Immune Cell Abundance Identifier (ImmuCellAI). They demonstrated that T cells and myeloid cells account for a large proportion of atherosclerotic immune cells and that macrophages and dendritic cells (DCs) revealed differential enrichments in the CAPs and control groups^13^. By using WGCNA, Zhao et al. detected an age-associated gene as a biomarker of CAPs , and in vitro experiments suggest that the gene *CEBPB* participates in the progression of CAPs^33^. The aforementioned studies aimed to describe the change in the abundance of 24 immune cell types in atherosclerotic tissues; however, unlike previous research, we aimed to identify differential genes associated with the change in the abundance of immune cells.

The present study is the first to combine the ssGSEA, Wilcoxon test, LASSO regression model, and WGCNA to identify the key infiltrating immune cells and genes between the CAPs and control groups, and it proposes immune infiltration-related biomarkers for CAPs.

By analyzing the immune characteristics of CAPs, we found significant differences for 11 immune cells (activated B cells, memory B cells, gamma-delta T cells, natural killer T cells, activated CD8 + T cells, central memory CD8 + T cells, effector memory CD8 + T cells, effector memory CD4 + T cells, natural killer cells, eosinophils, and monocytes) between the CAPs and control groups, thus suggesting that innate and adaptive immune systems play a pivotal role in driving chronic inflammation associated with CAPs in the arterial wall. Cytotoxic T cells (CD8 + T cells), Natural Killer T (NKT) cells, NK cells, and gamma-delta T cells are among the most important types of killer cells^34,35^. All the killer lymphocytes are implicated in the CAPs process through different methods such as cytotoxin-, FasL/TRAIL-, and/or cytokine-dependent mechanisms that eventually completes the process of rupture of CAPs^36,37^. Killer cells are abundant in unstable plaques, which reinforce their role in the rupture of CAPs and their involvement in the progression of carotid artery atherosclerosis. The different types of B cells (B1 and B2) exhibit disparate effects on the development of CAPs. For example, B1 cells produce IgM antibodies and show an atheroprotective effect, while B2 cells secrete IgG and IgE antibodies, which promote the development of CAPs^38^. Therefore, we speculate that immunity is closely related to CAPs and may affect the immunotherapeutic effects in patients with CAPs through killer lymphocyte-related mechanisms.

Among the key genes screened by MCODE on the basis of DEG co-expression network, the genes *LYN* and *BTK* showed the most remarkable connectivity. With a high average functional similarity, the genes *ITGAL* and *ITGB7* may play a vital role in the interaction network. The ROC curve of these genes showed that the AUC values were 0.854, 0.852, 0.851, 0.843, 0.844, 0.827, and 0.825 for *LYN*, *BTK*, *PTPN11*, *CD4*, *CD163*, *ITGAL*, and *ITGB7*, respectively.

LYN, a member of protein tyrosine kinases, primarily manages fundamental cellular processes such as cell growth and differentiation through the inhibition of immune activation by recruiting inhibitory proteins and lipid phosphatases^39^. Previous studies have shown that LYN regulates both positive and negative pathways in B cell-related immunity^40^. LYN is also a crucial regulator of immune receptor signaling pathways, and it promotes both proinflammatory and suppressive signaling pathways in immune cells (e.g., neutrophils, DCs, monocytes, and macrophages) and B lymphocytes^41^. This also agrees with the results of the present study that the 11 differential immune cells are mainly B lymphocytes and innate immune cells.

BTK is required to activate pathways that promote lymphocyte survival^42^ and to regulate the secretion of chemokines and B cell adhesion procedure by activating phospholipase Cg2 (PLCg2)^43^. Additionally, BTK appears to play a key role in innate immunity by modulating many immune signaling networks of the innate immune system^44^. BTK is also important for neutrophil development^45^ and for NLRP3-inflammasome activation in macrophages^46^ and DCs^47^. We also found that the genes *BTK* and *LYN* had the highest expression similarity at 0.94, thus indicating that both these genes are likely to exert the same effect on the development of CAPs.

CD4 is a membrane glycoprotein expressed on helper T lymphocytes, and it interacts with immunoglobulin molecules of nearly all classes and subclasses^48^. Infiltration of T lymphocytes has been found in sclerotic plaques at various stages of CAPs, among which CD4 + effector T lymphocytes are the most important ones^49,50^. CD4 + T cells are the core mediators of CAPs and participate in all stages of atherosclerotic diseases. Chemokine receptors (e.g., CCR5 and CXCR6) mediate the infiltration of T cells into the CAPs^51,52^.

CD163 is expressed exclusively in monocytes (low expression) and macrophages (high expression)^53^. IL-6, IL-10, and other anti-inflammatory cytokines induce CD163 expression, while IL-4, TNF-α, IFN-γ and other inflammatory factors inhibit CD163 expression^54^. It seems apparent that only a subpopulation of M2s is CD163 + ; hence, CD163 is often used as an M2 marker^55^. High expression of CD163 in macrophages is a feature of inflammatory tissues. Previous studies have shown that glucocorticoids exert anti-inflammatory effects on macrophages by affecting their phenotype and thus regulating the expression of cytokines^56^.

Protein tyrosine phosphatase N11 (PTPN11), also known as Src homology 2 domain-containing protein tyrosine phosphatase 2 (SHP2), is an important protein tyrosine phosphatase (PTP) that plays an integral role in multiple cellular events, including cell migration, differentiation, survival, and metabolism^57^. Chen J et al. found that the inhibition of the activity of SHP2 could prevent the development of CAPs by inhibiting the proliferation of vascular smooth muscle cells^58^.

Integrin β7 (ITGB7) is expressed on the surface of leukocytes, and it plays an essential role in the homing of immune cells^59^^.^ Previous studies have shown that ITGB7 is constitutively activated in multiple myeloma (MM) cells, and it has a remarkable anti-MM effect^60^. ITGB7 binds to integrin α4 to form integrin α4β7, which is a heterodimeric cell surface receptor expressed on most leukocytes^61^. Integrin α4β7 is involved in several steps during atherogenesis in mouse, and the upregulation of integrin α4β7 is associated with the progression of atherogenic lesions^62^.

Lymphocyte function-associated antigen-1, also named LFA-1 or αLβ2, is a pivotal T cell integrin to regulate T cell activation and migration^63^. However, to date, no studies have reported that ITGAL is directly relevant to the progression of CAPs. Previous studies have shown that ITGAL affects foam cell formation in macrophages by regulating Ac-LDL uptake^64^.

The results of DO enrichment analysis of the diagnostic genes showed that these genes were significantly enriched in tuberculosis, human immunodeficiency virus infectious disease, and primary immunodeficiency disease. CD4 is the receptor of the HIV envelope protein gp120; thus, HIV can selectively infect CD4 + T cells to cause AIDS. Previous studies suggest that the phenotype of macrophage populations may largely influence tuberculosis progression and infection outcome during the period from early infection to granuloma formation^65^. The M2 phenotype includes multiple forms of non-classically activated macrophages with anti-inflammatory, angiogenic, and Th2-directed immunomodulatory properties. Previous studies have concluded that during active tuberculosis infection, human monocytes tend to differentiate into anti-inflammatory (M2-like) macrophages that show enhanced protease-dependent motility, pathogen survival, and immunomodulatory properties^66^.

In the present study, we also used the DGIdb to screen several small molecule drugs with potential therapeutic efficacy against CAPs. Some of them, such as imatinib, have been proven to have anticancer effects for treating chronic myeloid leukemia and gastrointestinal stromal tumors^67^. Previous studies have also shown that imatinib can decrease and normalize hyperlipidemia, thus indicating that imatinib may attenuate the development of CAPs. Thus, these identified small molecule drugs might have significant potential to inhibit the progression of CAPs^68^.

The present study has some limitations. First, the use of three samples to validate the ROC analysis is inadequate. Second, because of the small number of datasets that were appropriate for this study, we selected only microarray datasets instead of RNA-seq and single-molecule sequencing datasets. Our future research will focus on closing the gap in this area. Third, we analyzed only the genes that might be related to CAPs, without in-depth analysis of cell subtype localization. We plan to conduct a new study that will enroll more patients and multiple types of datasets to verify the reliability of the hub genes.

In summary, we identified 7 immuno-related differential genes between CAPs and normal carotid artery samples for the first time, which included *BTK*, *LYN*, *PTPN11*, *CD163*, *CD4*, *ITGAL*, and ITGB7; this finding might be instrumental in understanding the process of CAPs and serve as potential targets of immunotherapy. In the future, we will further verify the accuracy of our results through relevant experiments on clinical tissue samples.

## Data Availability

The datasets are available from the GEO database. GSE43292 (https://www.ncbi.nlm.nih.gov/geo/query/acc.cgi), GSE100927 (https://www.ncbi.nlm.nih.gov/geo/query/acc.cgi).
